# Integrated cancer cell-specific single-cell RNA-seq datasets of immune checkpoint blockade-treated patients

**DOI:** 10.1038/s41597-025-04381-6

**Published:** 2025-01-22

**Authors:** Mahnoor N. Gondal, Marcin Cieslik, Arul M. Chinnaiyan

**Affiliations:** 1 Department of Computational Medicine & Bioinformatics, University of Michigan, Ann Arbor, MI USA; 2 Michigan Center for Translational Pathology, University of Michigan, Ann Arbor, MI USA; 3 Department of Pathology, University of Michigan, Ann Arbor, MI USA; 4 University of Michigan Rogel Cancer Center, Ann Arbor, MI USA; 5 Department of Urology, University of Michigan, Ann Arbor, MI USA; 6 Howard Hughes Medical Institute, Ann Arbor, MI USA

**Keywords:** Cancer immunotherapy, Computational biology and bioinformatics

## Abstract

Immune checkpoint blockade (ICB) therapies have emerged as a promising avenue for the treatment of various cancers. Despite their success, the efficacy of these treatments is variable across patients and cancer types. Numerous single-cell RNA-sequencing (scRNA-seq) studies have been conducted to unravel cell-specific responses to ICB treatment. However, these studies are limited in their sample sizes and require advanced coding skills for exploration. Here, we have compiled eight scRNA-seq datasets from nine cancer types, encompassing 223 patients, 90,270 cancer cells, and 265,671 other cell types. This compilation forms a unique resource tailored to investigate how cancer cells respond to ICB treatment across cancer types. We meticulously curated, quality-checked, pre-processed, and analyzed the data, ensuring easy access for researchers. Moreover, we designed a user-friendly interface for seamless exploration. By sharing the code and data for creating these interfaces, we aim to assist fellow researchers. These resources offer valuable support to those interested in leveraging and exploring single-cell datasets across diverse cancer types, facilitating a comprehensive understanding of ICB responses.

## Background & Summary

The cytotoxic activity of T cells can be deactivated through interaction between checkpoint proteins such as PD-1 found on the surface of T cells, and their counterparts, like PD-L1 or PD-L1, present on cancerous cells^1^. As a result, many cancer cells hijack this mechanism by overexpressing ligands such as PD-L1, disrupting the body’s immune response and impeding the T cells’ ability to eradicate cancer cells^1^. Research into understanding cancer immune evasion led to the development of immune checkpoint blockade (ICB) therapy that includes monoclonal antibodies that block checkpoint proteins’ functions, allowing activated cytotoxic T cells to eliminate cancer cells^2–4^. While such therapies have shown promise in treating different cancer types, their efficacy varies among individual patients and specific cancer types^5–14^. Numerous studies have attempted to uncover the factors contributing to the success or failure of ICB therapies, predominantly employing RNA expression data^15–21^. However, due to the multifactorial intrinsic properties of cancer cells, conventional bulk RNA sequencing lacks the resolution needed for an in-depth exploration of cancer cell-specific responses to ICB treatment.

Single-cell RNA-sequencing (scRNA-seq) provides a unique opportunity to dissect the intricate landscape of cancer cell-specific responses to ICB treatment. Numerous scRNA-seq studies have explored patients treated with ICB across various cancer types, including skin cancers such as melanoma^22–25^, and basal cell carcinoma^26^. Additionally, investigations have extended to breast cancer subtypes like triple negative^27^, HER2-positive^27^, ER-positive^27^, as well as kidney cancer, specifically clear cell renal carcinoma^28^, and liver cancers such as hepatocellular carcinoma^29^, intrahepatic cholangiocarcinoma^29^. Single-cell studies investigating ICB-treated patients have provided valuable insights into the cellular responses to immune checkpoint blockade therapies, potentially unveiling novel biomarkers^30^. However, these studies often are limited in their sample size and the inherent heterogeneity within tumors and among patients can make it challenging to generalize findings across a broader population. Additionally, single-cell data require sophisticated computational methods due to the large volume of data generated, posing challenges for researchers lacking extensive bioinformatics expertise. Therefore, to overcome limited sample sizes and the complexity of data analysis there is a pressing need to collate, standardize, aggregate, and deposit scRNA-seq datasets with ICB-treated patients to facilitate convenient exploration, ensuring accessibility for both wet and dry lab experts.

Here, we conducted an extensive literature review and meticulously performed data curation, quality control, pre-processing, and analysis. This effort resulted in a robust data resource comprising eight scRNA-seq datasets spanning nine distinct cancer types, involving 223 patients, 90,270 cancer cells, and 265,671 other cell types. To enhance accessibility, we have developed user-friendly interfaces housing single-cell data. To create standardized data across various studies, we employed normalization techniques using a gene signature known as stably expressed genes (hSEGs)^31^. Additionally, to evaluate cell-to-cell interactions between malignant cells and other cell types we have also incorporated other cell types in our resource. Through our efforts, users can effortlessly explore standardized datasets involving all cell types via https://scrnaseqicb.shinyapps.io/09_shinycell_all/ and for cancer-cell specific exploration https://scrnaseqicb.shinyapps.io/shinyapp/. This effort provides an unprecedented data resource tailored for investigating ICB responses in cancer cells across a wide range of cancer malignancies. The entire resource can also be accessed through the CZ CELLxGENE Discover website at https://cellxgene.cziscience.com/collections/61e422dd-c9cd-460e-9b91-72d9517348ef. Notably, our resource has been utilized in recent study^12^ to explore *PIKFYve* gene expressions in responders and non-responders within single-cell melanoma datasets, underscoring the versatility of our data in advancing research on ICB responses in various cancer types. In summary, these resources serve as a support for those keen on leveraging and delving into cancer cell-specific exploration across diverse cancer types, fostering a comprehensive exploration of ICB responses.

## Methods

**Fig. 1:**
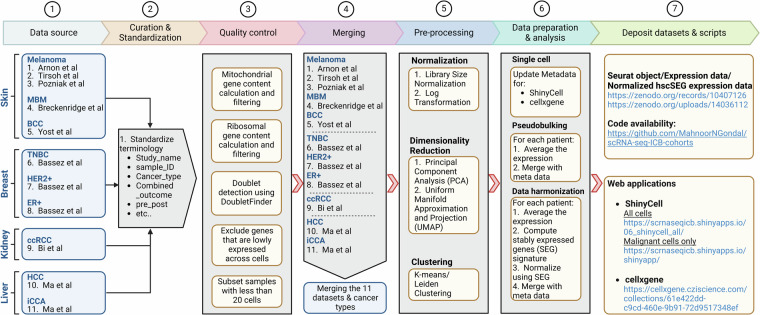
Workflow for developing a dedicated resource centered on cancer cells in ICB studies using single-cell data. The steps include conducting a comprehensive literature review of scRNA-seq studies, standardizing dataset terminologies, implementing rigorous quality control and pre-processing measures, merging datasets for subsequent analysis, and depositing resultant datasets alongside associated R scripts to ensure easy accessibility.

To construct a comprehensive data repository of single-cell RNA sequencing data (scRNA-seq) aimed at investigating cancer cell behavior under ICB treatment, we systematically executed seven key steps, elaborated upon in the following sections (Fig. 1).

### Data sources

To compile scRNA-seq datasets that detail cancer cell annotations in the context of ICB treatment, an extensive literature survey was undertaken. This survey identified eight key studies, specifically: Jerby-Arnon *et al*.^22^, Tirsoh *et al*.^23^, Pozniak *et al*.^25^, Yost *et al*.^26^, Alvarez-Breckenridge *et al*.^24^, Bassez *et al*.^27^, Bi *et al*.^28^, Ma *et al*.^29^. These studies encompassed various cancer types, including skin cancers such as melanoma^22,23,25^, basal cell carcinoma^26^, melanoma brain metastases^24^, as well as breast cancer, subtypes triple negative^27^, HER2-positive^27^, ER-positive^27^. Additionally, the studies covered kidney cancer (clear cell renal carcinoma^28^), and liver cancers like hepatocellular carcinoma^29^, and intrahepatic cholangiocarcinoma^29^. The information was extracted from diverse data resources and websites. A comprehensive breakdown of the original data sources and sample specifics is provided in Table 1.

**Table 1:** Details of data sources and information.

#	PMID	Authors	Technology	Cancer type	Cancer subtype	# of malignant cells	# of non-malignant cells	# of patients or samples (mal)	# of patients or samples (other)	Pre/UT (mal)	Pre/UT (other)	Post (mal)	Post (other)	Pre/Post (same p)	Primary/Meta	Location of original data	License/Data Use Agreement	DOI’s
1	30388455	Jerby-Arnon *et al.*	SmartSeq2	Skin	Melanoma	665	844	8	13	3 (UT)	6 (UT)	5 (Resis)	7 (Resis)	No	Both	[1-2]	publicly available	10.1016/j.cell.2018.09.006
2	27124452	Tirsoh *et al.*	SmartSeq2			602	1829	7	13	5 (UT)	6 (UT)	2 (Resis)	7 (Resis)	No	Met			
3	38181739	Pozniak *et al.*	10X			9201	0	28	0	17 (6 R, 11 NR)	0	11 (2 R, 9 NR)	0	No	Both	[3]	CC-BY-NC-SA-4.0	10.1016/j.cell.2023.11.037
4	35706413	Alvarez-Breckenridge *et al.*	SmartSeq2		MBM	4990	5905	24	25	8 (1 R, 2 NR, 5 UT)	9 (1 R, 2 NR, 6 UT)	16 (10 PR, 6 NR)	16 (11 PR, 5 NR)	No	Met	[4]	publicly available	10.1158/2326-6066.CIR-21-0870
5	31359002	Yost *et al.*	10X		BCC	3452	73077	8	28	4 [UT]	14 [6 R, 7 NR, 1 CR]	4 [2R, 2 NR]	14 [6 R, 7 NR, 1 CR]	No	Met	[5]	publicly available	10.1038/s41591-019-0522-3
6	31588021	Lichun Ma *et al.*	10X	Liver	HCC	743	4408	6	9	1	2	5	7	No	Both	[6]	publicly available	10.1016/j.ccell.2019.08.007
7					iCCA	1025	2509	8	10	1	1	7	9	No	Both			
8	33958794	Ayse Bassez *et al.*	10X	Breast	TNBC	32501	80532	36	38	18 [8 E, 9 NE, 1 n/a]	19 [8 E, 10 NE, 1 n/a]	18 [8 E, 9 NE, 1 n/a]	19 [8 E, 10 NE, 1 n/a]	Yes	Pri	[7]	publicly available	10.1038/s41591-021-01323-8
9					HER2	3108	12909	8	8	4 [1 E, 2 NE, 1 n/a]	4 [1 E, 2 NE, 1 n/a]	4 [1 E, 2 NE, 1 n/a]	4 [1 E, 2 NE, 1 n/a]	Yes	Pri			
10					ER+	27927	59721	35	38	18 [3 E, 15 NE]	19 [3 E, 16 NE]	17 [2 E, 15 NE]	19 [3 E, 16 NE]	Yes	Pi			
11	33711272	Bi *et al.*	10X	Kidney	ccRCC	6056	23937	6	7	3 [UT]	3 [UT]	3 [2 PR, 1 SD]	4 [2 PR, 1 SD, 1 NE]	No	Met	[8]	publicly available	10.1016/j.ccell.2021.02.015

This table presents metadata from eight studies, detailing original publication information, the count of malignant and other cell types, the number of patients involved, pre-and post-treatment statuses, original data sources, and the locations of processed files.

[1-2] - Jerby-Arnon + Tirsoh - https://www.ncbi.nlm.nih.gov/geo/query/acc.cgi?acc=GSE115978.

[3] - Pozniak - https://rdr.kuleuven.be/dataset.xhtml?persistentId=doi:10.48804/GSAXBN.

[4] - Alvarez-Breckenridge - https://singlecell.broadinstitute.org/single_cell/study/SCP1493/microenvironmental-correlates-of-immune-checkpoint-inhibitor-response-in-human-melanoma-brain-metastases-revealed-by-t-cell-receptor-and-single-cell-rna-sequencing#study-download.

[5] - Yost - https://www.ncbi.nlm.nih.gov/geo/query/acc.cgi?acc=GSE123813.

[6] - Ma - https://www.ncbi.nlm.nih.gov/geo/query/acc.cgi?acc=GSE125449.

[7] - Bassez - https://lambrechtslab.sites.vib.be/en/single-cell.

[8] - Bi - https://singlecell.broadinstitute.org/single_cell/study/SCP1288/tumor-and-immune-reprogramming-during-immunotherapy-in-advanced-renal-cell-carcinoma#study-download.

### Data curation and standardization

**Fig. 2:**
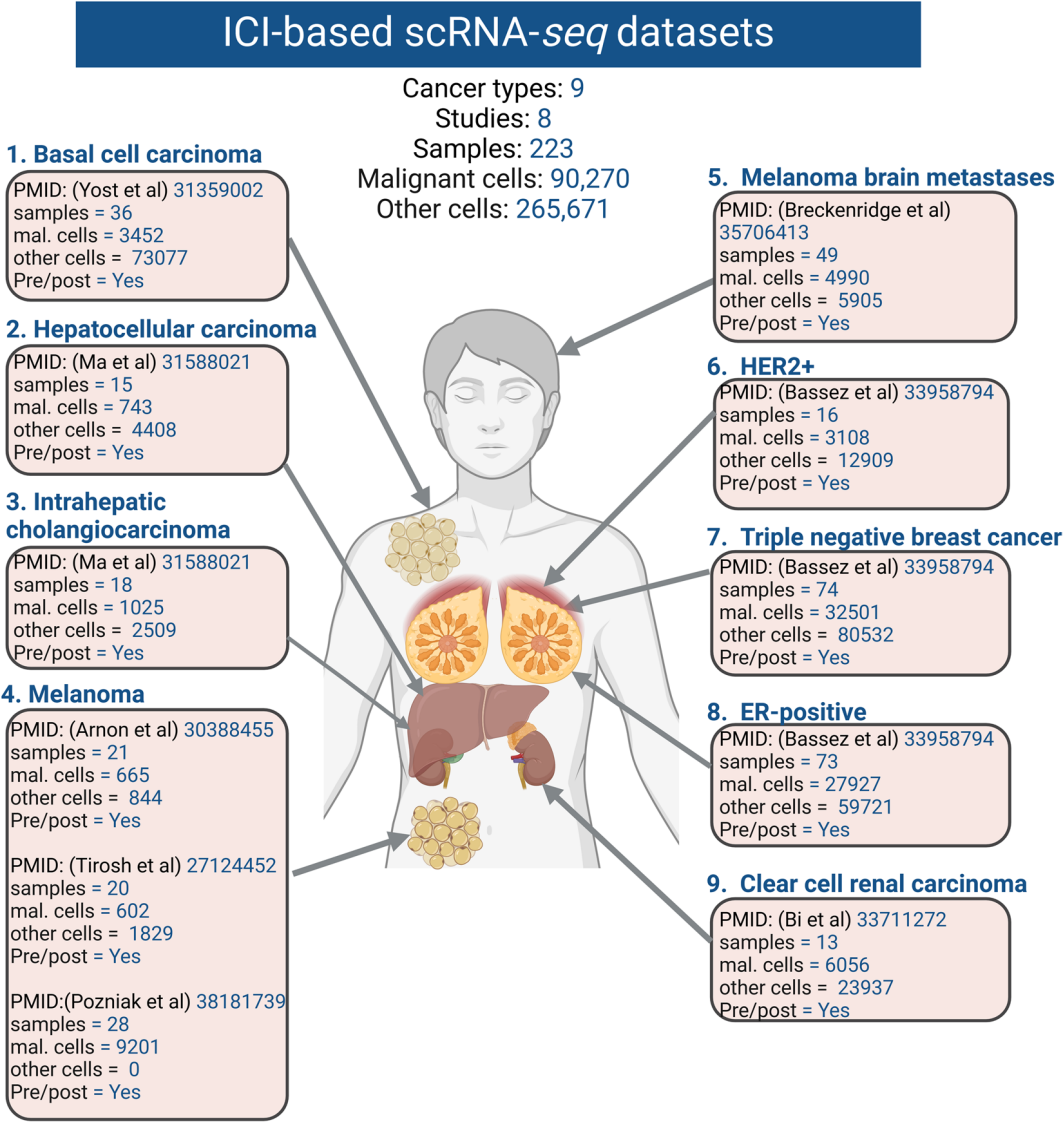
Overview of single-cell studies investigated in this resource. The basal cell carcinoma dataset comprised 3,452 cancer cells and 73,077 non-malignant cells, while the hepatocellular carcinoma dataset contained 743 cancer cells and 4,408 non-malignant cells, and the intrahepatic cholangiocarcinoma dataset included 1,025 cells and 2,509 non-malignant cells. The combined three melanoma datasets yielded a total of 10,468 malignant cells and 2,673 non-malignant cells, with the melanoma brain metastasis dataset contributing 4,990 cancer cells and 5,905 non-malignant cells. Within the breast cancer datasets, there were 3,108 (HER2+), 32,501 (TNBC), and 27,927 (ER+) cancer cells, and 12,909 (HER2+), 80,532 (TNBC), and 59,721 (ER+) non-malignant cells, respectively. Additionally, the clear cell carcinoma dataset comprised 6,056 cancer cells and 23,937 non-malignant cells. In total, the resulting dataset amalgamates findings from eight studies encompassing nine distinct cancer types, encapsulating a sum of 90,270 malignant cells and 265,671 non-malignant cells.

We performed meticulous data extraction, curation, and standardization for each individual single-cell study, drawing from diverse resources and databases. Acknowledging potential discrepancies arising from varying naming conventions and cell annotations among different authors, we anticipated discrepancies. To maintain uniformity and coherence within our resources, we took steps to standardize terminologies across all studies before proceeding (Fig. 2). Additional specifics for each study are provided below for clarity.

#### Jerby-Arnon *et al*. and Tirsoh *et al*. (Melanoma)

Jerby-Arnon *et al*.^22^ unveiled a cancer cell mechanism fostering T cell exclusion, contributing to resistance against checkpoint blockade therapies. This program elucidated the mechanisms behind the evasion of immune surveillance in tumors, impacting the effectiveness of immunotherapy. In contrast, Tirosh *et al*.^23^ leveraged scRNA-seq to unravel the complex multicellular landscape of metastatic melanoma. It comprehensively profiled individual cells within the tumor microenvironment, offering insights into cellular interactions, heterogeneity, and potential therapeutic targets for this aggressive cancer.

The Jerby-Arnon datasets for melanoma samples were extracted from a publicly available site eg GEO under GSE115978 (https://www.ncbi.nlm.nih.gov/geo/query/acc.cgi?acc=GSE115978). The study builds upon the previously published melanoma study by Tirsoh *et al*. and as a result, already contained the previous samples. Therefore we standardized both Jerby-Arnon *et al*. and Tirosh *et al*.‘s melanoma studies together. The metadata was also supplied from the Tumor Immune Single Cell Hub (TISCH)^32^. The data was first converted to a Seurat object using raw counts data, and cell annotation files. The samples were renamed to “sample_id”. As mentioned in the original publication, all treated patients were classified as resistant to ICB treatment and categorized as “Unfavourable” in the “Combined_outcome” criteria. InferCNV was employed to identify malignant cells in both cohorts. Access to the processed Seurat object in the RDS file and the associated Rmd file for this dataset has been provided for accessibility (Table 2).

**Table 2:** Summary of code and data-related Items.

	Objects	Description	Datasets	File name	Location
**Malignant Cells Only**	**RDS objects**	**RDS objects for each study without quality control**	**BCC** (**Yost**)	seurat_BCC_SCC_tumor_subset_subset.RDS	https://zenodo.org/records/10407126
			**Breast** (**Bassez**)	seurat_Bassez_counts_treatment_naive_neoadjuvant_epi_subset.RDS	
			**Kidney** (**Bi**)	Bi_seurat_md_na_tumor_subset_ccRCC.RDS	
			**Liver** (**Ma**)	seurat_Liver_seurat_set_1_2_epi_subset.RDS	
			**MBM** (**Alvarez**)	seurat_MBM_Christopher_m_epi_subset.RDS	
			**Melanoma** (**Arnon + Tirosh**)	seurat_Arnon_data_metadata_M_mel_subset2.RDS	
			**Melanoma** (**Pozniak**)	seurat_melanoma_joanna.RDS	
			**Combined data**	merged_filtered_tissue_cluster_sig-003.RDS	
	**CSV Files**	**Downsampled single cell data**	**Single cell objects**	Rshinydata_singlecell-20231219T155916Z-001.zip	
		**Pseudobulk datasets for each study**	**Pseudobulks objects**	Rshinydata_pseudobulk-20231219T155911Z-001.zip	
		**Harmonized data together - Pseudobulk**	**Combined_datasets**	Rshinydata_hscSEG_normalized_data-20231219T155901Z-001.zip	
		**Individual harmonized datasets - Pseudobulk**	**Individual_datasets**		
	**Rmd files**	**Rmd file for the combined analysis involving step 3 to 7 steps of the workflow**	**Combined data**	Pipeline_ICB_Combined_26.Rmd	https://github.com/MahnoorNGondal/scRNA-seq-ICB-cohorts/tree/main/R/Combined_results
		**Individual R scripts for each study involving data curation,** **Seurat object creation,** **and extraction of cancer cells**	**MBM** (**Alvarez**)	Pipeline_MBM_Breckenridge.Rmd	https://github.com/MahnoorNGondal/scRNA-seq-ICB-cohorts/tree/main/R/01_MBM_Breckenridge
			**Breast** (**Bassez**)	Pipeline_Breast_Bassez (1).Rmd	https://github.com/MahnoorNGondal/scRNA-seq-ICB-cohorts/tree/main/R/02_Breast_Bassez
			**Kidney** (**Bi**)	Pipeline_Kidney_Bi.Rmd	https://github.com/MahnoorNGondal/scRNA-seq-ICB-cohorts/tree/main/R/03_Kidney_Bi
			**Melanoma** (**Pozniak**)	Pipeline_Melanoma_Pozniak.Rmd	https://github.com/MahnoorNGondal/scRNA-seq-ICB-cohorts/tree/main/R/04_Melanoma_Pozniak
			**BCC** (**Yost**)	Pipeline_BCC_Yost.Rmd	https://github.com/MahnoorNGondal/scRNA-seq-ICB-cohorts/tree/main/R/05_BCC_Yost
			**Liver** (**Ma**)	Pipeline_Liver_Ma.Rmd	https://github.com/MahnoorNGondal/scRNA-seq-ICB-cohorts/tree/main/R/06_Liver_Ma
			**Melanoma** (**Arnon + Tirosh**)	Pipeline_melanoma_Arnon&Tirosh.Rmd	https://github.com/MahnoorNGondal/scRNA-seq-ICB-cohorts/tree/main/R/07_08_Melanoma_Jerby-Arnon_%26_Tirosh
	**Rshiny applications**	**Rshiny application for downsampled single cell data**	**Rshiny_pseudobulk**	app.R	https://github.com/MahnoorNGondal/scRNA-seq-ICB-cohorts/tree/main/R/Rshiny_singlecell
		**Rshiny application for pseudobulk data**	**Rshiny_singlecell**	app.R	https://github.com/MahnoorNGondal/scRNA-seq-ICB-cohorts/tree/main/R/Rshiny_pseudobulk
**All Cells Types**	**RDS objects**	**RDS objects for each study without quality control**	**Breast** (**Bassez**)	All_Celltypes_seurat_Bassez_counts_treatment_naive_neoadjuvant_epi_subset.RDS	https://drive.google.com/file/d/1ZFte8Cs5k59diuOtj_9W0LngNxTXq99O/view?usp = sharing
			**BCC** (**Yost**)	All_Celltypes_seurat_BCC_SCC_tumor_subset_subset.RDS	https://zenodo.org/records/14511579
			**Kidney** (**Bi**)	All_Celltypes_Bi_seurat_md_na_tumor_subset_ccRCC.RDS	
			**Liver** (**Ma**)	All_Celltypes_seurat_Liver_seurat_set_1_2_epi_subset.RDS	
			**MBM** (**Alvarez**)	All_Celltypes_seurat_MBM_Christopher_m_epi_subset.RDS	
			**Melanoma** (**Arnon + Tirosh**)	All_Celltypes_seurat_Arnon_data_metadata_M_mel_subset2.RDS	
			**Melanoma** (**Pozniak**)	All_Celltypes_seurat_melanoma_joanna.RDS	
			**Combined data**	merged_tissue_cluster_update_filt_other_mal_4.RDS	
	**CSV Files**	**Downsampled single cell data**	**Single cell objects**	All_Celltypes_Rshinydata_singlecell-20241105T203605Z.zip	
		**Pseudobulk datasets for each study**	**Pseudobulks objects**	All_Celltypes_Rshinydata_pseudobulk-20241105T203601Z-001.zip	
		**Harmonized data together - Pseudobulk**	**Combined_datasets**	All_Celltypes_Rshinydata_hscSEG_normalized_data-20241105T203546Z-001.zip	
		**Individual harmonized datasets - Pseudobulk**	**Individual_datasets**		
	**Rmd files**	**Rmd file for the combined analysis involving step 3 to 7 steps of the workflow**	**Combined data**	Pipeline_ICB_Combined_42_revised_all_celltypes.Rmd	https://github.com/MahnoorNGondal/scRNA-seq-ICB-cohorts/tree/main/R/Combined_results
		**Individual R scripts for each study involving data curation,** **Seurat object creation,** **and extraction of cancer cells**	**MBM** (**Alvarez**)	Pipeline_MBM_Breckenridge_revised_all_celltypes.Rmd	https://github.com/MahnoorNGondal/scRNA-seq-ICB-cohorts/tree/main/R/01_MBM_Breckenridge
			**Breast** (**Bassez**)	Pipeline_Breast_Bassez_revised_all_celltypes.Rmd	https://github.com/MahnoorNGondal/scRNA-seq-ICB-cohorts/tree/main/R/02_Breast_Bassez
			**Kidney** (**Bi**)	Pipeline_Kidney_Bi_revised_all_celltypes.Rmd	https://github.com/MahnoorNGondal/scRNA-seq-ICB-cohorts/tree/main/R/03_Kidney_Bi
			**Melanoma** (**Pozniak**)	Pipeline_Melanoma_Pozniak.Rmd	https://github.com/MahnoorNGondal/scRNA-seq-ICB-cohorts/tree/main/R/04_Melanoma_Pozniak
			**BCC** (**Yost**)	Pipeline_BCC_Yost_revised_all_celltypes.Rmd	https://github.com/MahnoorNGondal/scRNA-seq-ICB-cohorts/tree/main/R/05_BCC_Yost
			**Liver** (**Ma**)	Pipeline_Liver_Ma_revised_all_celltypes.Rmd	https://github.com/MahnoorNGondal/scRNA-seq-ICB-cohorts/tree/main/R/06_Liver_Ma
			**Melanoma** (**Arnon + Tirosh**)	Pipeline_melanoma_Arnon&Tirosh_revised_all_celltypes.Rmd	https://github.com/MahnoorNGondal/scRNA-seq-ICB-cohorts/tree/main/R/07_08_Melanoma_Jerby-Arnon_%26_Tirosh

This table offers a comprehensive overview, encompassing file types, detailed descriptions, names, and locations, including the type of data. Its purpose is to streamline accessibility and serve as a convenient point of reference for users seeking access to code and data resources.

#### Pozniak *et al*. (Melanoma)

A recently published melanoma scRNA-seq study by Pozniak *et al*.^25^ was utilized in our resource. This study highlighted the significant impact of intrinsic resistance on ICB effectiveness in melanoma. By analyzing scRNA-seq data from patients undergoing ICB therapy, the study identified TCF4 as a key regulator of the Mesenchymal-like (MES) program, suggesting a potential therapeutic avenue to enhance immunogenicity in ICB-resistant melanomas and improve sensitivity to targeted therapy.

The dataset from this study was publicly accessible through KU Leuven Research Data Repository (RDR) under the license CC-BY-NC-SA-4.0 in the form of RDS files with metadata included (https://rdr.kuleuven.be/dataset.xhtml?persistentId=doi:10.48804/GSAXBN). This was already a Seurat object with raw count data included as well as the clinical information for both responders and non-responders. Responders were categorized as “Favourable” and non-responders as “Unfavourable” in the “Combined_outcome” criteria. Inferred CNV profiles based on scRNA-seq data were employed to identify malignant cells using the HoneyBADGER method^33^. The processed Seurat object in the RDS file and the associated Rmd file for this dataset are made accessible (Table 2).

#### Yost *et al*. (Basal Cell Carcinoma)

Yost *et al*.^26^ study, revealed the phenomenon of clonal replacement where new tumor-specific T cell clones emerged after PD-1 blockade treatment in Basal Cell Carcinoma (BCC). This replacement suggested a dynamic shift in the T cell population, potentially impacting the efficacy of PD-1 blockade therapy in the past.

scRNA-seq data for this study was publicly available on GSE123814, alongside metadata information (https://www.ncbi.nlm.nih.gov/geo/query/acc.cgi?acc=GSE123813). Raw counts and metadata were employed to create a Seurat object. The dataset also contained squamous cell carcinoma (SCC) samples, however, since SCC lacked malignant cells we removed it from the final dataset. Single-cell CNVs were detected to label malignant cells using HoneyBADGER^33^. Those with outcomes “Yes” were labeled as “Favourable” and those with “No” were categorized as “Unfavorable” in the “Combined_outcome” criteria. The processed Seurat object in the RDS file, along with the associated Rmd file for this dataset, is now accessible (Table 2).

#### Alvarez-Breckenridge *et al*. (Melanoma Brain Metastasis)

Melanoma brain metastases (MBM) refer to the spread of malignant melanoma cells from their primary site to the brain, forming secondary tumors. These metastases present significant challenges due to their aggressive nature, often leading to severe neurological complications and impacting treatment options. Alvarez-Brechenridge *et al*.^24^ explored the microenvironmental changes in human melanoma brain metastases triggered by immune checkpoint inhibition. The study provided insights into the alterations within the brain metastatic sites in response to immune checkpoint therapy, shedding light on the intricate interplay between the tumor microenvironment and treatment efficacy.

This dataset was accessible using Broad Institute’s single-cell portal (a publicly available site) (https://singlecell.broadinstitute.org/single_cell/study/SCP1493/microenvironmental-correlates-of-immune-checkpoint-inhibitor-response-in-human-melanoma-brain-metastases-revealed-by-t-cell-receptor-and-single-cell-rna-sequencing#study-download). We downloaded the raw count data from the portal alongside the barcode and features matrix to create the Seurat object. The metadata was later added using the AddMetaData function in the Seurat package. Malignant cells were labeled using inferred copy-number profiles within cells, from a single patient, using all FACS gating categories (CD45−, CD45+, CD3+). The resultant copy number profiles were then projected onto their first principal component towards generating a “malignancy score” which was later utilized for malignant cell detection. Responders and partial-responders were categorized as “Favourable” and non-responders as “Unfavourable” in the “Combined_outcome” criteria. The processed Seurat object stored in the RDS file, along with the associated Rmd file for this dataset, is available for access (Table 2).

#### Bassez *et al*. (HER2+, ER+, Triple-negative Breast Cancer)

The Bassez *et al*.^27^ study created a single-cell map, detailing intratumoral alterations occurring during anti-PD1 treatment in breast cancer patients (TNBC, HER2+, ER+). It provided insights into the dynamic changes within the tumor microenvironment in response to anti-PD1 therapy, aiding in understanding treatment effects and potential therapeutic avenues.

This dataset encompassed two cohorts; Cohort 1 contained treatment-naive samples while Cohort 2 had neo-adjuvant therapy samples. Datasets including the two cohorts’ counts and metadata are easily and publically accessible through the VIB-KU Leuven Center for Cancer Biology website (https://lambrechtslab.sites.vib.be/en/single-cell). Both cohorts were first converted to Seurat objects and then combined for downstream analysis. InferCNV was employed to identify copy number alterations in the cells to label malignant cells in both cohorts. The clinical outcomes in this study were divided between “expanded” and “non-expanded”. As such, expanded samples were categorized as “Favourable” and non-expanded as “Unfavourable” in the “Combined_outcome” criteria. The Seurat object, processed and stored in the RDS file, as well as the corresponding Rmd file for this dataset, are both accessible (Table 2).

#### Bi *et al*. (Clear Cell Renal Carcinoma)

Bi *et al*.^28^ study documented tumor and immune system reprogramming occurring during immunotherapy for renal cell carcinoma patients. It offered insights into the dynamic changes within both the tumor and immune cells, shedding light on the mechanisms involved in immunotherapy response and resistance.

The dataset was downloaded from Curated Cancer Cell Atlas (3CA; https://www.weizmann.ac.il/sites/3CA/kidney) database^34^ and single cell broad institute portal (publicly available sites) (https://singlecell.broadinstitute.org/single_cell/study/SCP1288/tumor-and-immune-reprogramming-during-immunotherapy-in-advanced-renal-cell-carcinoma#study-download) including the raw counts, features, and barcodes alongside patients’ clinical information. These were employed to create a Seurat object. Inferred Copy Number Aberration (infercna) (using the package available at https://github.com/jlaffy/infercna) was utilized to identify malignant cells. Partial-responders were categorized as “Favourable” and stable disease as “Unfavourable” in the “Combined_outcome” criteria. The processed Seurat object, encapsulated within the RDS file, and its related Rmd file for this dataset have been made readily available for access and utilization (Table 2).

#### Ma *et al*. (Hepatocellular Carcinoma, Intrahepatic Cholangiocarcinoma)

Ma *et al*.‘s^29^ research illustrated that the variability within liver cancer cells played a significant role in reshaping the tumor microenvironment. It emphasized how the diversity among cancer cells impacted the changes occurring in the surrounding tumor environment, offering a critical understanding of the intricate relationship between cancer cells and their immediate surroundings.

Both Hepatocellular carcinoma (HCC) and intrahepatic cholangiocarcinoma (iCCA) datasets were downloaded from a publicly available site eg GEO GSE125449 including raw counts, barcodes, and features to create two Seurat objects (https://www.ncbi.nlm.nih.gov/geo/query/acc.cgi?acc=GSE125449). The two Seurat objects were then merged. InferCNV was employed to identify malignant cells in both cohorts. The processed Seurat object stored within the RDS file and the associated Rmd file for this dataset are now accessible for utilization and reference purposes (Table 2).

### Quality control

The quality control steps are described in detail under technical validation.

### Merging

For subsequent analysis, we used Seurat’s merge function to combine the eight datasets. The resulting merged data comprises 223 patients and 90,270 cancer cells and 265,671 non-malignant cells, spanning nine cancer types.

### Pre-processing

**Fig. 3:**
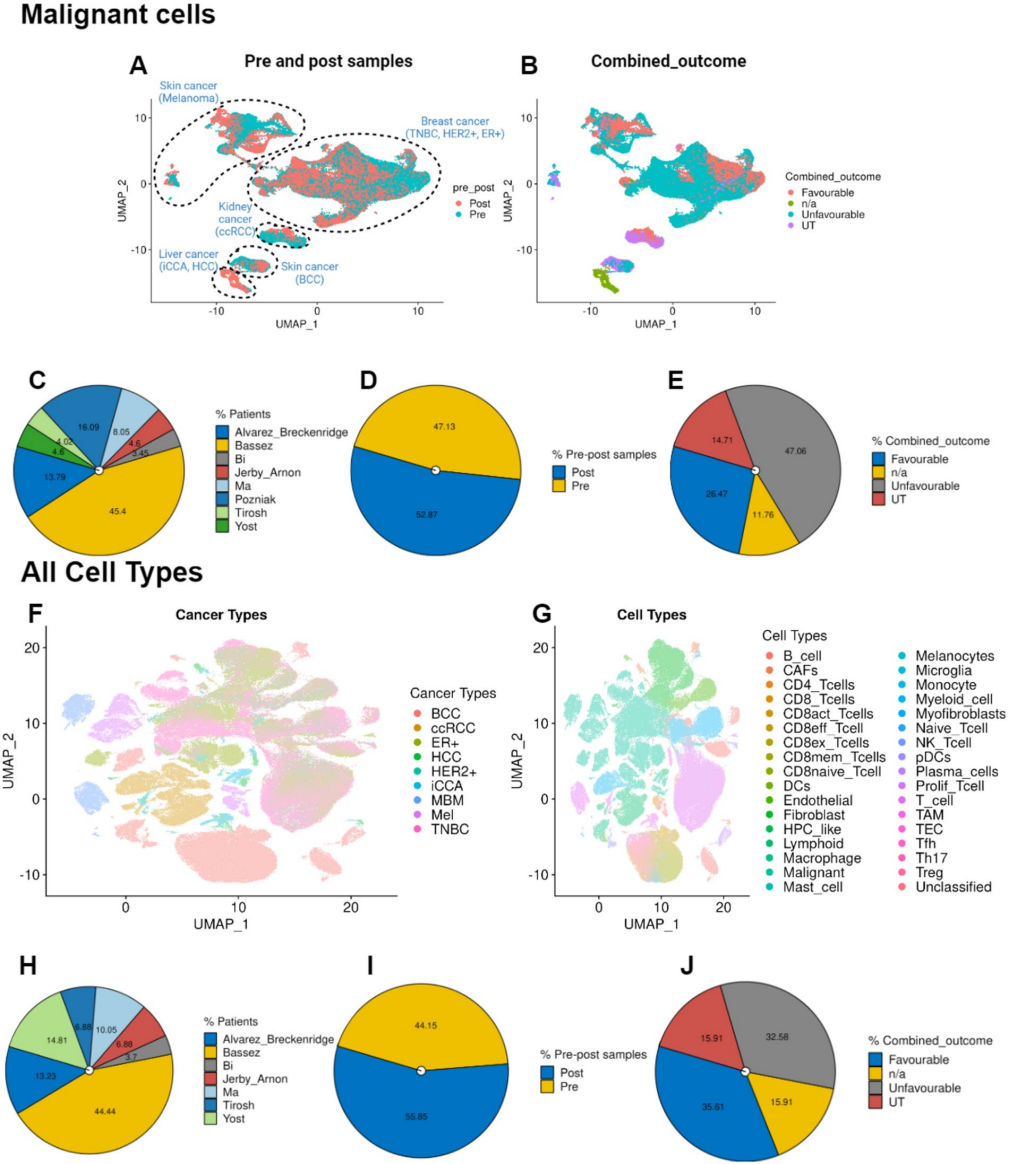
Visualization of data distribution across studies in malignant cells only and in other cell types. (**A**) UMAP visualizations depicting pre-and post-treatment status, and cancer type information in malignant cells only. (**B**) UMAP for combined outcome distributions within different datasets in malignant cells. (**C**–**E**) Pie charts illustrating patient distribution across studies, total pre- and post-treatment samples, and ‘Favourable,’ ‘Unfavourable,’ ‘Untreated’ (UT), and ‘n/a,’ combined outcomes across studies in the merged dataset in malignant cells only. (**A**) UMAP depicting cancer-type information across all cell types. (**B**) UMAP for displaying cell types in the data resource. (**H**–**J**) Pie charts illustrating patient distribution across studies, total pre- and post-treatment samples, and ‘Favourable,’ ‘Unfavourable,’ ‘Untreated’ (UT), and ‘n/a,’ combined outcomes across studies in the merged dataset in non-malignant or other cell types.

Single-cell analysis was performed using the Seurat package (v4.1.1). To pre-process the datasets we employed Seurat’s standardized pipeline. Data normalization was performed using log1p normalization from Seurat’s NormalizeData function. Dimensionality reduction and clustering were undertaken using an unsupervised graph-based clustering approach using 20 dimensions. Uniform Manifold Approximation and Projection (UMAP) was employed to visualize the data. For the malignant cells, the UMAP was visualized using individual cancer types across pre and post-samples (Fig. 3A). We also observed UMAP in light of ‘Favourable,’ ‘Unfavourable,’ ‘Untreated’ (UT), and ‘n/a,’ combined outcomes across both study and cancer types (Fig. 3B). Detailed sample and study information is available in Table 1. Furthermore, a comprehensive overview of the datasets, including the number of samples per study, sample sizes in pre and post-datasets, and cancer cells within ‘Combined_outcomes,’ is presented in Fig. 3C–E. Similarly, for all cell types, we have visualized the data in the form of UMAPs for individual cancer types as well as cell types presented in this data resource (Fig. 3F,G). Additionally, we also provide a comprehensive overview of the datasets, including the number of samples per study, sample sizes in pre and post-datasets, and non-malignant cells within ‘Combined_outcomes,’ is presented in Fig. 3H–J.

### Data preparation and analysis

We have made the Seurat objects available for use in the form of RDS files the details of which are provided in Table 2. Analyzing single-cell data demands advanced computational methods owing to the vast amount of generated data, presenting challenges for researchers lacking deep bioinformatics knowledge. To facilitate accessibility for non-bioinformaticians, we deposited CSV files necessary for data exploration as well as developed Rshiny applications using the ShinyCell package^35^. Web applications are a user-friendly tool that provides access to distinct data types within the application interface. Further specifics on the data generation process are outlined below.

#### Cancer-cell-specific single-cell data

To analyze the cancer-cell-specific single-cell RNAseq datasets within the ShinyCell application, we initiated by subseting to cancer cells only from each sample in both pre and post-treatment contexts. Following this, we employed the ShinyCell package to develop the R shiny application with relevant metadata. The ShinyCell application can be found here: https://scrnaseqicb.shinyapps.io/shinyapp/. The code associated with the application can be found on our GitHub. For further exploration by the users, the files can be downloaded directly using Zenodo (10.5281/zenodo.10407125)^36^.

#### All cell types in single-cell data

To evaluate all cell types in the single-cell RNAseq datasets within the ShinyCell application, we initiated by employing the ShinyCell package to develop a web application and then deployed this application to rsconnect server. The ShinyCell application can be found here: https://scrnaseqicb.shinyapps.io/09_shinycell_all/. These generated files are also downloaded as RDS formats and archived on Zenodo and for further exploration by the users, the files can be downloaded directly using Zenodo (10.5281/zenodo.10407125)^36^.

#### Modifications for CZ CELLxGENE discover database

Additionally, we also processed data to deposit on the CZ CELLxGENE Discover database. For this, we updated the metadata as requested by the CZ CELLxGENE Discover team following the guidelines on their website for data deposition. This data can be accessed here: https://cellxgene.cziscience.com/collections/61e422dd-c9cd-460e-9b91-72d9517348ef.

#### Generation and analysis of pseudobulk data

To manage extensive datasets and circumvent zero values in the raw count for single-cell data, we generated pseudobulks for individual samples in both pre and post-treatment settings. This involved subsetting to each study and employing Seurat’s AverageExpression function to extract the pre and post-cluster expression of all genes. Subsequently, this combined data, integrated with study metadata, was downloaded in CSV format and archived on Zenodo (10.5281/zenodo.10407125). The pseudobulk data involving malignant or cancer cells can be found here: https://zenodo.org/records/10407126^36^, whereas the pseudobulk data with other individual cell types as well can be accessed here: https://zenodo.org/records/14511579^36^. The processed data stored within the CSV file for this dataset are now accessible for utilization and reference purposes (Table 2).

#### Harmonization of single-cell data

Single-cell cohorts often face limitations due to sample size. To collectively analyze these cohorts, we normalized the datasets by initially segmenting them into each study. Using the AverageExpression function, we created pseudobulks for pre and post-samples as well as cell types. Next, we employed stably expressed genes (hSEGs) gene signature^31^ to normalize within each cohort, effectively correcting for batch effects. Each dataset was then combined into one and archived on Zenodo in a combined and individual structure (10.5281/zenodo.10407125). The harmonized data for malignant cells can be accessed here: https://zenodo.org/records/10407126^36^. Additionally, the harmonized data including other cell types is available here: https://zenodo.org/records/14511579^36^. The processed data for this dataset is now available in a CSV file for use and reference (see Table 2).

### Deposit datasets and scripts

Finally, we have prioritized accessibility by ensuring the R script’s availability to the users. We achieved this by archiving the scripts on GitHub, to encourage collaboration, version control, and users to engage with and contribute to the codebase. Additionally, we developed user-friendly Rshiny applications, providing an intuitive interface for efficient data interaction. Furthermore, to enable users to easily access and reference the data, we shared the data in the form of CSV and RDS files on Zenodo (10.5281/zenodo.10407125), fostering an open and collaborative environment, details provided in Table 2.

## Data Records

The data files are available for download in CSV and RDS formats from the Zenodo data platform (10.5281/zenodo.10407125)^36^. You can find the R Markdown scripts on GitHub at https://github.com/MahnoorNGondal/scRNA-seq-ICB-cohorts. The web applications are accessible at https://scrnaseqicb.shinyapps.io/09_shinycell_all/ for all cell types and https://scrnaseqicb.shinyapps.io/shinyapp/ for cancer cell-specific analysis. Additionally, this data can also be accessed via the CZ CELLxGENE Discover database at https://cellxgene.cziscience.com/collections/61e422dd-c9cd-460e-9b91-72d9517348ef.

The individual files are described in Table 2.

## Technical Validation

**Fig. 4:**
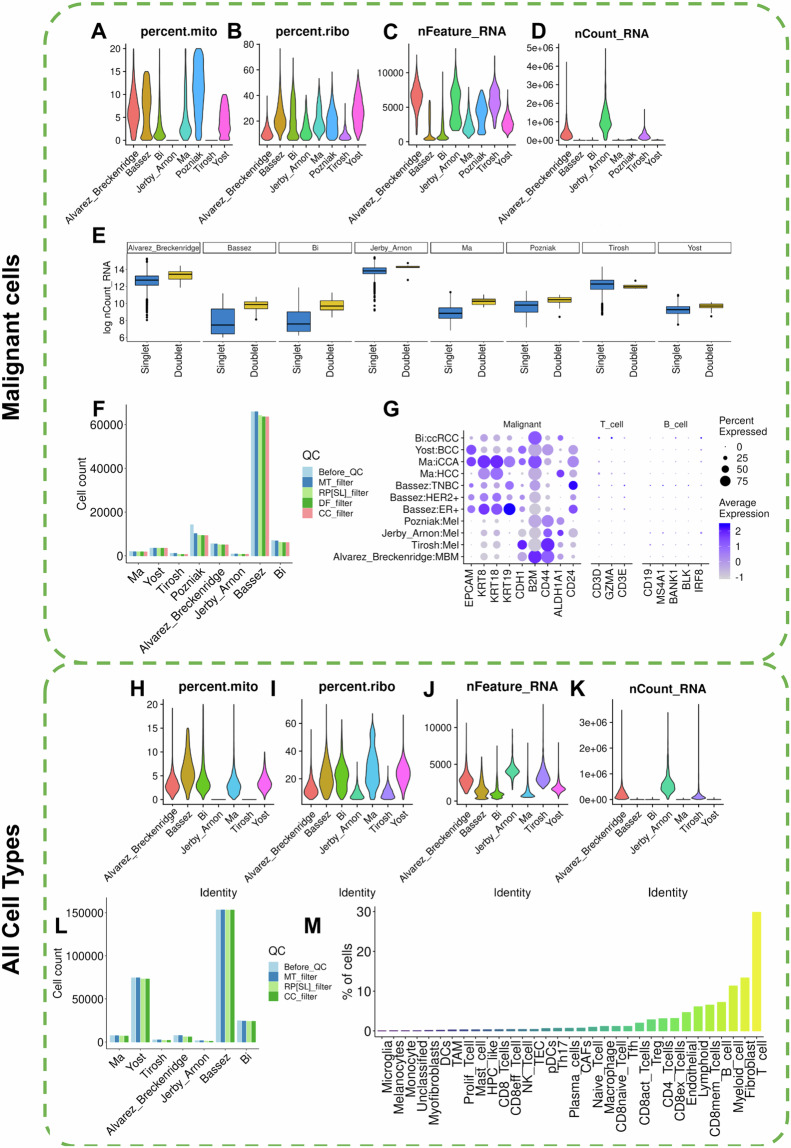
Quality control and technical validation across studies. (**A**–**D**) Outline the data subsetting process, retaining cells with less than 20% mitochondrial gene content, ensuring genes are expressed in at least 3 cells, and retaining cells with a ribosomal gene content of at least 5%. (**E**) Demonstrates the execution of DoubletFinder, filtering the dataset to retain only ‘Singlet’ cells. (**F**) visually represents the number of cancer cells retained at each filtering step (Before_QC: before quality control, MT_filter: mitochondrial gene content filter, RP[SL]_filter: ribosomal gene content filter, DF_filter: DoubletFinder filter, CC_filter: cancer cells per patient filter). (**G**) showcases highlighted markers specific to epithelial (malignant) cells ensuring data quality. (**H**–**K**) Details similar quality control steps applied to non-malignant cell types, following the same mitochondrial and ribosomal gene content filters. (**L**) Breakdown of the number of non-malignant cells retained at each quality control step. (**M**) The final dataset of non-malignant cells illustrates the overall reduction after filtering.

For each study, a comprehensive series of quality control measures were systematically implemented to ensure the robustness of the single-cell data. The original dataset encompassed a total of 100,025 cancer cells. To safeguard data quality, the first step involved the identification and removal of cells exhibiting high mitochondrial content, which is indicative of potentially compromised or dead cells^37^. The percentage of mitochondrial gene content was calculated for each cell using the PercentageFeatureSet function. Cells surpassing a 20% threshold in mitochondrial gene content were excluded, resulting in the removal of 4.48% of cells (Fig. 4A).

Subsequently, to enhance the quality of the dataset, an additional criterion was applied, focusing on ribosomal gene content. Cells with less than 5% ribosomal RNA expression were excluded using the same function, leading to the retention of 91,320 cancer cells (Fig. 4B). Further refinement involved filtering the dataset to retain only genes expressed in a minimum of 4 cells (Fig. 4C,D).

To address potential technical issues arising from doublets^37^ (instances of more than one cell per bead), the DoubletFinder^38^ algorithm was employed with default parameters for each study. This process identified and eliminated ‘Doublets,’ or low-quality cells leaving only ‘Singlet’ cells and resulting in 90,407 cancer cells (Fig. 4E).

Additional refinement steps included ensuring that each patient’s data contained a minimum of 20 malignant cells. Figure 4F breaks down the number of cells at each step of the quality control process. The final datasets, after these quality control steps, comprised 90,270 cancer cells, reflecting the removal of 9.75% of the initial cells.

Validation of the data was conducted using established markers for malignant (epithelial), T cells, and B cells. Remarkably, the validation process underscored markers exclusively associated with malignant cells (Fig. 4G), affirming the quality and specificity of the refined datasets.

Following the validation of malignant cells, we performed similar quality control procedures for the non-malignant cell types. We began with 277,860 cells and applied the same initial filtering steps, starting with the removal of cells with a high percentage of mitochondrial gene content. An additional filter based on ribosomal gene content was then applied, resulting in the retention of 266,153 cells (Fig. 4H–K). Further refinement ensured that each patient’s dataset included a minimum of 20 cells from each non-malignant cell type. The number of cells at each stage of quality control is shown in Fig. 4J. After these steps, 265,671 cells remained, representing a 4.39% reduction from the initial cell count (Fig. 4M).

## Data Availability

The code can be accessed on GitHub, https://github.com/MahnoorNGondal/scRNA-seq-ICB-cohorts. All packages and their versions are mentioned on GitHub. The datasets can be assessed freely from zenodo (10.5281/zenodo.10407125)^36^.

## References

[1] Latchman, Y. *et al*. PD-L2 is a second ligand for PD-1 and inhibits T cell activation. *Nat. Immunol.* **2**, 261–268 (2001). 11224527 10.1038/85330

[2] Topalian, S. L., Taube, J. M. & Pardoll, D. M. Neoadjuvant checkpoint blockade for cancer immunotherapy. *Science* **367**, (2020). 10.1126/science.aax0182 PMC7789854 32001626

[3] Gondal, M. N. *et al*. Abstract 860: Pan-tissue master regulator inference reveals mechanisms of MHC alterations in cancers. *Cancer Res.* **84**, 860–860 (2024).

[4] Gondal, M. N. *et al*. TISON: a next-generation multi-scale modeling theatre for in silico systems oncology. *Systems Biology* (2021).

[5] Hodi, F. S. *et al*. Improved survival with ipilimumab in patients with metastatic melanoma. *N. Engl. J. Med.* **363**, 711–723 (2010). 20525992 10.1056/NEJMoa1003466 PMC3549297

[6] Robert, C. *et al*. Pembrolizumab versus ipilimumab in advanced melanoma (KEYNOTE-006): post-hoc 5-year results from an open-label, multicentre, randomised, controlled, phase 3 study. *Lancet Oncol.* **20**, 1239–1251 (2019). 31345627 10.1016/S1470-2045(19)30388-2

[7] Wolchok, J. D. *et al*. Nivolumab plus Ipilimumab in Advanced Melanoma. *N. Engl. J. Med.* **369**, 122–133 (2013). 23724867 10.1056/NEJMoa1302369 PMC5698004

[8] Garon, E. B. *et al*. Pembrolizumab for the Treatment of Non–Small-Cell Lung Cancer. *N. Engl. J. Med.* **372**, 2018–2028 (2015). 25891174 10.1056/NEJMoa1501824

[9] Rittmeyer, A. *et al*. Atezolizumab versus docetaxel in patients with previously treated non-small-cell lung cancer (OAK): a phase 3, open-label, multicentre randomised controlled trial. *Lancet* **389**, 255–265 (2017). 27979383 10.1016/S0140-6736(16)32517-X PMC6886121

[10] Davila, M. L. *et al*. Efficacy and toxicity management of 19-28z CAR T cell therapy in B cell acute lymphoblastic leukemia. *Sci. Transl. Med.* **6**, 224ra25 (2014). 24553386 10.1126/scitranslmed.3008226 PMC4684949

[11] Fyfe, G. *et al*. Results of treatment of 255 patients with metastatic renal cell carcinoma who received high-dose recombinant interleukin-2 therapy. *J. Clin. Oncol.* **13**, 688–696 (1995). 7884429 10.1200/JCO.1995.13.3.688

[12] Bao, Y. *et al*. Targeting the lipid kinase PIKfyve upregulates surface expression of MHC class I to augment cancer immunotherapy. *Proc. Natl. Acad. Sci. USA.* **120**, e2314416120 (2023). 38011559 10.1073/pnas.2314416120 PMC10710078

[13] Choi, J. E. *et al*. PIKfyve, expressed by CD11c-positive cells, controls tumor immunity. *Nat. Commun.* **15**, 5487 (2024). 38942798 10.1038/s41467-024-48931-9 PMC11213953

[14] Gondal, M. N. *et al*. A personalized therapeutics approach using an in silico Drosophila Patient Model reveals optimal chemo- and targeted therapy combinations for colorectal cancer. *Front. Oncol.* **11**, 692592 (2021). 34336681 10.3389/fonc.2021.692592 PMC8323493

[15] Gide, T. N. *et al*. Distinct Immune Cell Populations Define Response to Anti-PD-1 Monotherapy and Anti-PD-1/Anti-CTLA-4 Combined Therapy. *Cancer Cell* **35**, 238–255.e6 (2019). 30753825 10.1016/j.ccell.2019.01.003

[16] Riaz, N. *et al*. Tumor and Microenvironment Evolution during Immunotherapy with Nivolumab. *Cell* **171**, 934–949.e16 (2017). 29033130 10.1016/j.cell.2017.09.028 PMC5685550

[17] Van Allen, E. M. *et al*. Genomic correlates of response to CTLA-4 blockade in metastatic melanoma. *Science* **350**, 207–211 (2015). 26359337 10.1126/science.aad0095 PMC5054517

[18] Hugo, W. *et al*. Genomic and Transcriptomic Features of Response to Anti-PD-1 Therapy in Metastatic Melanoma. *Cell* **168**, 542 (2017). 28129544 10.1016/j.cell.2017.01.010

[19] Mariathasan, S. *et al*. TGFβ attenuates tumour response to PD-L1 blockade by contributing to exclusion of T cells. *Nature* **554**, 544–548 (2018). 29443960 10.1038/nature25501 PMC6028240

[20] Miao, D. *et al*. Genomic correlates of response to immune checkpoint therapies in clear cell renal cell carcinoma. *Science* **359**, 801–806 (2018). 29301960 10.1126/science.aan5951 PMC6035749

[21] Gondal, M. N. & Chaudhary, S. U. Navigating Multi-Scale Cancer Systems Biology Towards Model-Driven Clinical Oncology and Its Applications in Personalized Therapeutics. *Front. Oncol.* **11**, 712505 (2021). 34900668 10.3389/fonc.2021.712505 PMC8652070

[22] Jerby-Arnon, L. *et al*. A Cancer Cell Program Promotes T Cell Exclusion and Resistance to Checkpoint Blockade. *Cell* **175**, 984–997.e24 (2018). 30388455 10.1016/j.cell.2018.09.006 PMC6410377

[23] Tirosh, I. *et al*. Dissecting the multicellular ecosystem of metastatic melanoma by single-cell RNA-seq. *Science* **352**, 189–196 (2016). 27124452 10.1126/science.aad0501 PMC4944528

[24] Alvarez-Breckenridge, C. *et al*. Microenvironmental Landscape of Human Melanoma Brain Metastases in Response to Immune Checkpoint Inhibition. *Cancer Immunol Res* **10**, 996–1012 (2022). 35706413 10.1158/2326-6066.CIR-21-0870 PMC10201927

[25] Pozniak, J. *et al*. A TCF4-dependent gene regulatory network confers resistance to immunotherapy in melanoma. *Cell* **187**, 166–183.e25 (2024). 38181739 10.1016/j.cell.2023.11.037

[26] Yost, K. E. *et al*. Clonal replacement of tumor-specific T cells following PD-1 blockade. *Nat. Med.* **25**, 1251–1259 (2019). 31359002 10.1038/s41591-019-0522-3 PMC6689255

[27] Bassez, A. *et al*. A single-cell map of intratumoral changes during anti-PD1 treatment of patients with breast cancer. *Nat. Med.* **27**, 820–832 (2021). 33958794 10.1038/s41591-021-01323-8

[28] Bi, K. *et al*. Tumor and immune reprogramming during immunotherapy in advanced renal cell carcinoma. *Cancer Cell* **39**, 649–661.e5 (2021). 33711272 10.1016/j.ccell.2021.02.015 PMC8115394

[29] Ma, L. *et al*. Tumor Cell Biodiversity Drives Microenvironmental Reprogramming in Liver Cancer. *Cancer Cell* **36**, 418–430.e6 (2019). 31588021 10.1016/j.ccell.2019.08.007 PMC6801104

[30] Gondal, M. N., Shah, S. U. R., Chinnaiyan, A. M. & Cieslik, M. A systematic overview of single-cell transcriptomics databases, their use cases, and limitations. *Front. Bioinform.* **4**, 1417428 (2024). 39040140 10.3389/fbinf.2024.1417428 PMC11260681

[31] Lin, Y. *et al*. Evaluating stably expressed genes in single cells. *Gigascience* **8**, (2019). 10.1093/gigascience/giz106 PMC6748759 31531674

[32] Sun, D. *et al*. TISCH: a comprehensive web resource enabling interactive single-cell transcriptome visualization of tumor microenvironment. *Nucleic Acids Res.* **49**, D1420–D1430 (2021). 33179754 10.1093/nar/gkaa1020 PMC7778907

[33] Fan, J. *et al*. Linking transcriptional and genetic tumor heterogeneity through allele analysis of single-cell RNA-seq data. *Genome Res.* **28**, 1217–1227 (2018). 29898899 10.1101/gr.228080.117 PMC6071640

[34] Gavish, A. *et al*. Hallmarks of transcriptional intratumour heterogeneity across a thousand tumours. *Nature* **618**, 598–606 (2023). 37258682 10.1038/s41586-023-06130-4

[35] Ouyang, J. F., Kamaraj, U. S., Cao, E. Y. & Rackham, O. J. L. ShinyCell: simple and sharable visualization of single-cell gene expression data. *Bioinformatics* **37**, 3374–3376 (2021). 33774659 10.1093/bioinformatics/btab209

[36] Gondal, M., Cieslik, M. & Chinnaiyan, A. Integrated cancer cell-specific single-cell RNA-seq datasets of immune checkpoint blockade-treated patients. *Zenodo*10.5281/ZENODO.10407125 (2024). 10.1038/s41597-025-04381-6 PMC11754430 39843468

[37] Heumos, L. *et al*. Best practices for single-cell analysis across modalities. *Nat. Rev. Genet.* **24**, 550–572 (2023). 37002403 10.1038/s41576-023-00586-w PMC10066026

[38] McGinnis, C. S., Murrow, L. M. & Gartner, Z. J. DoubletFinder: Doublet Detection in Single-Cell RNA Sequencing Data Using Artificial Nearest Neighbors. *Cell Syst* **8**, 329–337.e4 (2019). 30954475 10.1016/j.cels.2019.03.003 PMC6853612

